# Spontaneous Hematoma Into the Carpal Tunnel and Palmar Aponeurosis

**Published:** 2015-01-23

**Authors:** Priyanka Chadha, Matthew Wordsworth, Rupert Eckersley

**Affiliations:** Chelsea and Westminster NHS Trust, London, UK

**Keywords:** hematoma, carpal tunnel, palmar aponeurosis, atraumatic, warfarin

**Figure 1:**
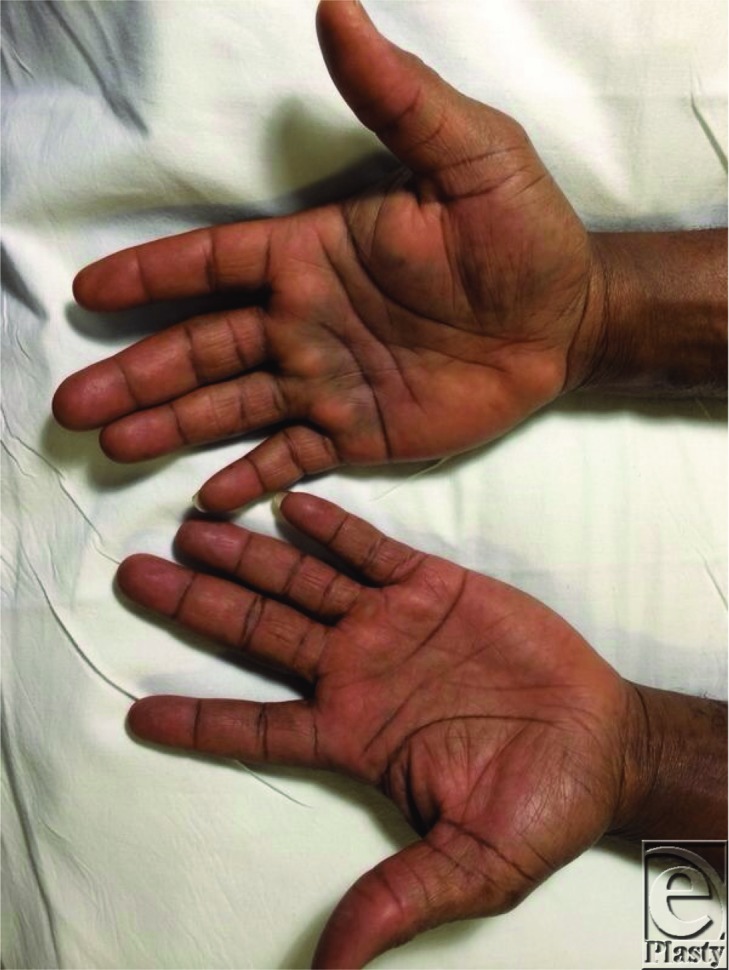


**Figure 2:**
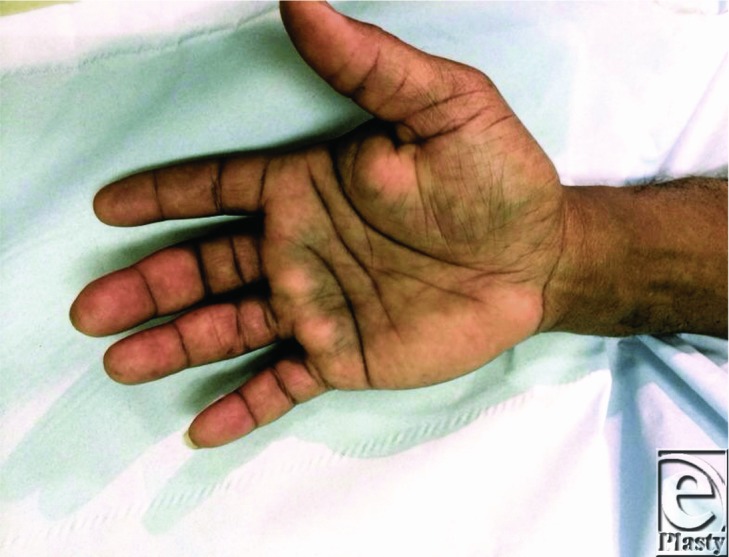


## DESCRIPTION

We describe the case of a 49-year-old gentleman who presented with an atraumatic, sudden onset of pain, over a 1-hour period, in his right hand accompanied by altered sensation to his middle, ring, and little fingers. On examination of his hand, there was an obvious bruise delineating the palmar aponeurosis. Ultrasonographic scan confirmed a hematoma in the distribution of the carpal tunnel and palmar aponeurosis, which was later attributed to excess anticoagulation from his warfarin medication.

## QUESTIONS

1. **What is the risk of spontaneous bleeding in a patient anticoagulated with warfarin?**
2. **How should the anticoagulation status be managed after the onset of symptoms?**
3. **What is the indication for surgical intervention?**
4. **What are the nonoperative treatments available?**

## DISCUSSION

A well-known complication of warfarin is that of spontaneous bleeding when the international normalized ratio (INR) is inappropriately high. For any patient, the potential benefit from prevention of thromboembolic disease must be weighed up against the potential harm from spontaneous hemorrhage.^1^ Hematomas can be minor or major in volume and clinical significance and varying incidence rates exist in the current literature, with the incidence of complications from major hemorrhage ranging from 1% to 3% per year and minor hemorrhage complication rates ranging from 4.8% to 9.5% per year.^2^ Documentation of a spontaneous hematoma in the colon, spine, and even rectus sheath can be found in the current literature.^3^ Patients have been known to present with a vast array of clinical signs and symptoms ranging from mild bruising to frank hematuria and hematemesis, requiring different levels of resuscitative care. However, an atraumatic, spontaneous hematoma into the carpal tunnel, palmar aponeurosis, and flexor sheath leading to symptoms from compression of the median nerve and ulnar nerves is not currently documented. The patient in this case presented with symptoms, which evolved over a 1-hour period, and on examination he had a positive Tinel's test and it was evident that his symptoms were mimicking that of carpal tunnel syndrome. His capillary refill time was 3 seconds and his fingers were bilaterally cool, with a palpable radial pulse. The patient had a medical history of lower limb thromboses and had been taking warfarin medication for many years. Coagulation blood tests revealed that his INR value was 4.0 and an ultrasonographic scan confirmed the diagnosis of a hematoma in his dominant hand.

Once the anticoagulation status of the patient has been assessed, it is essential to request specialist clinical input from the hematology team. For most patients, the offending medication should be stopped and cautiously reversed using intravenous Vitamin K (if the offending medication is warfarin) while seeking expert opinion. This decision should take into account the risks of thromboembolic disease against that of continued bleeding and evolving symptoms. Anticoagulation medication should be restarted when the INR value normalizes and the patient should be monitored after discharge in an anticoagulation clinic. Throughout the patient's hospital stay, it is essential to ensure that bleeding from other areas is not occurring by undertaking a thorough history and examination.

Operative management may be indicated if the clinical significance on presentation is urgent due to compression of surrounding nerves or suspected ischemia of the muscles from compartment syndrome. This can often be assessed clinically or through the direct measurement of compartment pressures. In addition, if the patient's symptoms do not improve after watchful waiting, then operative management for decompression of the limb and evacuation of the hematoma may be required. The patient should be regularly reviewed with repeat blood investigations and thorough documentation of clinical findings.

Nonoperative management options are also available for spontaneous hematomas. Often, as in this scenario, elevation of a limb, in combination with coagulation reversal, can reduce the swelling and prevent compartment syndrome and vascular compromise. Symptoms often resolve over 24 hours of close monitoring.

Spontaneous hematoma into the carpal tunnel and palmar aponeurosis causing median and ulnar nerve symptoms has not been previously documented in the literature. Ultrasonographic scan is a cost-effective and noninvasive method of diagnosing this condition and often nonsurgical management can resolve an isolated episode.
